# Pure Water Splitting Driven by Overlapping Electric Double Layers

**DOI:** 10.1021/jacs.4c01070

**Published:** 2024-07-10

**Authors:** Haosen Xu, Jianbo Zhang, Michael Eikerling, Jun Huang

**Affiliations:** † School of Vehicle and Mobility, State Key Laboratory of Intelligent Green Vehicle and Mobility, Tsinghua University, 100084 Beijing, China; ‡ IEK-13, Institute of Energy and Climate Research, Forschungszentrum Jülich GmbH, 52425 Jülich, Germany; § Chair of Theory and Computation of Energy Materials, Faculty of Georesources and Materials Engineering, RWTH Aachen University, 52062 Aachen, Germany; ∥ Theory of Electrocatalytic Interfaces, Faculty of Georesources and Materials Engineering, RWTH Aachen University, 52062 Aachen, Germany

## Abstract

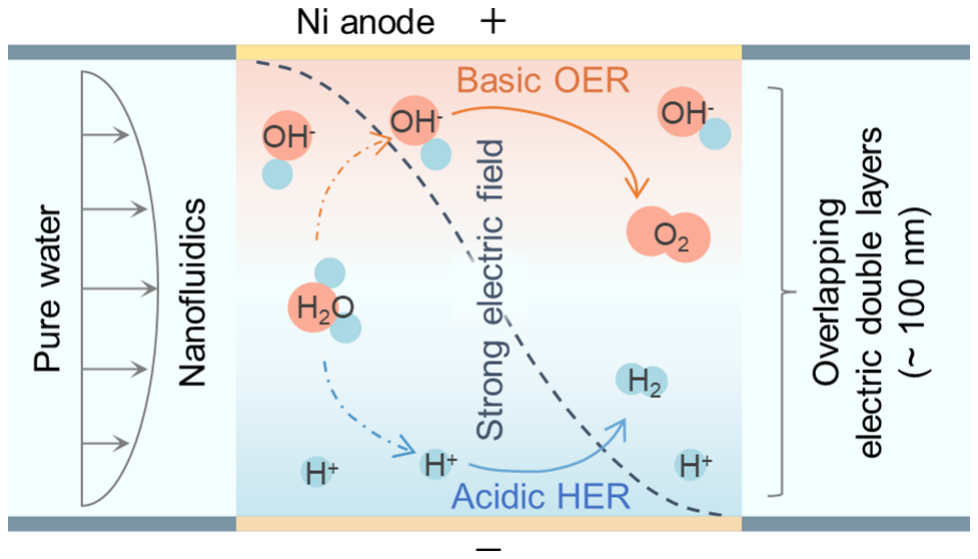

In pursuit of a sustainable future powered by renewable energy, hydrogen production through water splitting should achieve high energy efficiency with economical materials. Here, we present a nanofluidic electrolyzer that leverages overlapping cathode and anode electric double layers (EDLs) to drive the splitting of pure water. Convective flow is introduced between the nanogap electrodes to suppress the crossover of generated gases. The strong electric field within the overlapping EDLs enhances ion migration and facilitates the dissociation of water molecules. Acidic and basic environments, which are created *in situ* at the cathode and anode, respectively, enable the use of nonprecious metal catalysts. All these merits allow the reactor to exhibit a current density of 2.8 A·cm^–2^ at 1.7 V with a nickel anode. This paves the way toward a new type of water electrolyzer that needs no membrane, no supporting electrolyte, and no precious metal catalysts.

## Introduction

A defossilized global energy ecosystem hinges on deploying renewable energy sources like solar and wind power.^1^ Hydrogen (H_2_) emerges as a leading energy storage medium to mitigate the fluctuations and temporal–spatial distributions inherent in renewable energy harvesting.^2^ Powered by cheap electricity from renewable sources, electrochemical water splitting is a clean, affordable, and scalable way of green H_2_ production. However, insufficient energy efficiency and high material costs remain major obstacles to the large-scale deployment of water electrolysis. The challenge is further exacerbated by the fluctuating nature of renewable energy, which limits the operation time of water electrolyzers at full capacity.^3^

Despite significant progress in water electrolysis technologies, existing variants still fall short in meeting target metrics, either in terms of energy efficiency or cost-effectiveness. Alkaline water electrolysis, with simple configurations and low material costs, has attained a high readiness level,^4^ but needs a substantial increase in power density.^5−7^ Proton exchange membrane water electrolysis (PEMWE) achieves high efficiency, large current density, and superior dynamic responsiveness,^8−10^ but relies on expensive iridium catalysts to catalyze the sluggish acidic O_2_ evolution reaction (OER); moreover, it requires titanium porous transport layers to endure the harsh acidic anode environment.^11−14^ Emerging water electrolysis techniques are moving in two directions. In the pursuit of high power density, bipolar membrane water electrolysis (BPMWE) reduces the activation overpotentials by establishing an acidic environment for H_2_ evolution reaction (HER) and a basic environment for OER.^15−18^ Meanwhile, in efforts to reduce material cost, membranes or diaphragms are eliminated, via decoupled water splitting that segregates HER and OER in distinct steps,^19−22^ and membrane-less water electrolysis that exploits flow to separate the generated gases.^3,23−25^ However, a method that can achieve high efficiency at a practical current density with low material costs is currently lacking.

In this work, we adopt an unconventional principle of electrochemical water splitting driven by a strong electric field in the overlapping cathode and anode electric double layers (EDLs). We bring the interelectrode distance down below the characteristic length of EDLs, resulting in their strong overlap. A pervasive electric field with a strength exceeding 10^7^ V·m^–1^ can be established within the overlapping EDLs. This electric field can accelerate the migration of ions across the two electrodes^26^ and facilitate the dissociation of water molecules.^27^ The electric field enhancement effect has been investigated in fundamental kinetics studies^28,29^ and applied to electrochemical capacitors^30^ and sensors.^31−34^ In the field of water electrolysis, Wang et al. invented a nanogap laminated-type reactor with the overlapping EDLs.^35^ Their reactor with pure water can deliver a continuous current, which was attributed to pure water electrolysis enabled by the strong electric field in overlapping EDLs. However, the presence of the continuous current does not necessarily indicate the production of H_2_. Their design utilized a silicon-based intermediate layer to separate the cathode and anode electrodes, yet its surface was proved to be electrically conductive due to adsorbed ions in the presence of water and a strong electric field.^36,37^ Therefore, it is plausible that the observed current is caused by short-circuiting at the surface of the intermediate layer, rather than by water electrolysis. In addition, a crucial challenge arises from the unwanted crossover of H_2_ and O_2_ from one electrode to the other, reconverting H_2_ and O_2_ to water molecules. The gas crossover diminishes the current efficiency and could even lead to a catastrophic failure of H_2_ harvesting.

Here, we report a nanofluidic electrolyzer that splits pure water within the overlapping cathode and anode EDLs and effectively improves the current efficiency. Specifically, we introduce convection to the nanochannel between the two confronting electrodes to suppress the crossover of H_2_ and O_2_. A pair of detector electrodes is embedded downstream of the nanochannel to directly quantify the H_2_ output. The performance of this pure water splitting device is assessed as a function of interelectrode distance, electrode materials, water flow rate, and the concentration of the electrolyte solution. Mechanistic understanding of the overlapping EDLs is obtained using physical modeling. It is demonstrated that this unconventional electrolysis configuration yields state-of-the-art performance at much-reduced material costs, which offers a promising path to cheap green hydrogen. Aside from electrochemical water splitting, the overlapping EDLs show the potential as a general strategy to enhance ion transport and tune local reaction conditions.

## Results and Discussion

### Nanofluidic Reactor for Pure Water Splitting

**Figure 1:**
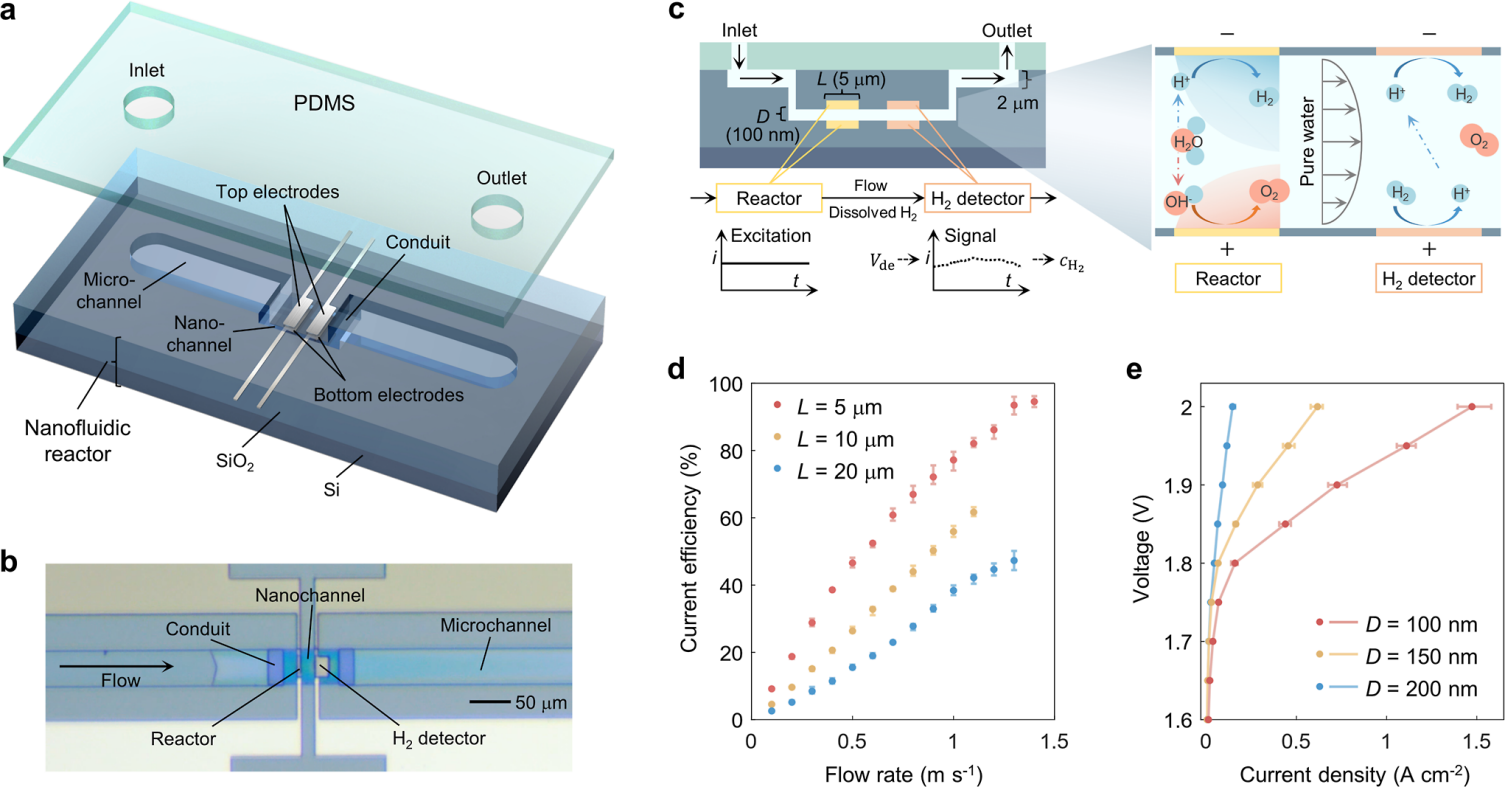
Nanofluidic reactor for pure water splitting with suppressed gas crossover. (a) Schematic diagram of the nanofluidic reactor. Fabricated in a SiO_2_ layer on a Si wafer, the nanofluidic reactor features two pairs of confronting platinum (Pt) electrodes. Each pair consists of two electrodes placed on the top and bottom sides of a nanochannel (100–200 nm in depth). Conduits (500 nm in depth) connect the nanochannel to microchannels (2 μm in depth). The upper ends of the microchannels are sealed by a cube of PDMS. (b) Top view of the reactor. A liquid water flow is pumped through the microchannel and conduit toward the nanochannel. (c) Cross-sectional side view of the reactor and a schematic diagram showing the respective functions of the two electrode pairs. The upstream confronting electrodes (reactor part) split water into H_2_ and O_2_, whose concentration distribution (illustrated by gradient colors) and transport are controlled by the rate of the convective flow. The downstream electrodes (H_2_ detector part) measure the current signal under a specific voltage (*V*_de_) to quantify the current efficiency. *D* represents the distance of the confronting electrode and the depth of the nanochannel, and *L* denotes the length of the reactor electrodes along the nanochannel. (d) The current efficiency exhibits a positive correlation with the flow rate. The current efficiency was measured at 2 V for the reactor and 0.4 V for the H_2_ detector, 20 °C and *D* = 200 nm. (e) The performance of pure water splitting improves with decreasing electrode distance. The current density is defined as the ratio of the current (0–5 μA) of the reactor to the overlapping area of the confronting electrodes (30 μm width times the length *L* of the reactor electrodes). The performance was tested at 60 °C, 1 m·s^–1^ flow rate and *L* = 5 μm, corrected by current efficiency. Notably, gases remained in the dissolved state during measurements of current efficiency and performance.

The nanofluidic reactor consists of two confronting electrodes positioned at a distance that corresponds to the depth of a straight nanochannel in between (Figure 1a). The nanochannel is interconnected with microchannels through conduits that bridge the gap between micrometer and nanometer scales (Figure 1b). This scale transition is key for providing convective flow to the nanochannel. In addition, the high pressure of the nanoscale convection presents a challenge to the mechanical integrity of the reactor. Ensuring the bonding strength between the chip and polydimethylsiloxane (PDMS), as well as the proper sealing of the water inlet, are essential to guarantee the functionality of the nanofluidic reactor. Detailed descriptions of the experimental procedures and parameters can be found in the Method Section of the Supporting Information. The water flow serves multiple functions, including supplying water, exporting the generated H_2_ and O_2_, and regulating the gas diffusion boundaries to inhibit the gas crossover (Figure 1c).

Detector electrodes are integrated downstream of the same nanochannel to quantify the amount of H_2_ output. This proof-of-principle-type design addresses the challenge of detecting dissolved H_2_ on the magnitude of picomolar in pure water. The current signal of the detector is amplified due to the reduced ohmic resistance and accelerated H^+^ migration from anode to cathode within the overlapping EDLs (Figure 1c). To address the mixing problem of O_2_ and H_2_, we meticulously select the voltage of the detector, at which H_2_ evolution and oxidation reaction occur at the cathode and anode, respectively, while the electrodes are inert to O_2_. We conducted linear sweep voltammetry experiments of the detector in pure water with dissolved H_2_, O_2_, and N_2_, individually. The current of H_2_ rises rapidly from 0 to 0.2 V and remains high thereafter, whereas O_2_ reactions occur only above 0.5 V (Figure S2). Thus, we selected 0.4 V as the voltage for the detector. At this voltage, we obtained a linear relationship between the current of the detector and the concentration of dissolved H_2_ at various flow rates (Figure S3). This calibrated relationship allows us to determine the amount of H_2_ that flows out of the reactor based on the current signal of the detector. Subsequently, we can calculate the current efficiency, which is defined as the ratio of the current corresponding to the output H_2_ to the total current of the reactor (see Supplementary Note 1 for more details about the calibration of the H_2_ detector and the calculation of the current efficiency).

Pure water splitting is realized using the nanofluidic reactor, with the production of H_2_ confirmed and the current efficiency quantified by the detector. The current efficiency rises from close to 0 to 95% as the flow rate increases for an electrode length of 5 μm (Figure 1d). A higher flow rate and a narrower electrode promote the escape of generated gases from the reactor, thus reducing gas crossover to the opposing electrodes. Consequently, after correction of the current efficiency, the current–voltage (IV) curves of pure water splitting are obtained at various distances of the electrodes (Figure 1e). A 10-fold improvement in the current density at a voltage of 2 V is obtained when the interelectrode distance decreases from 200 to 100 nm. This performance improvement cannot be attributed exclusively to the reduced ohmic resistance at the halved distance. More importantly, it is to be revealed that the performance improvement is caused by changes in ion transport and reaction environments as the overlap of cathode and anode EDLs increases.

### Effects of Overlapping EDLs

**Figure 2:**
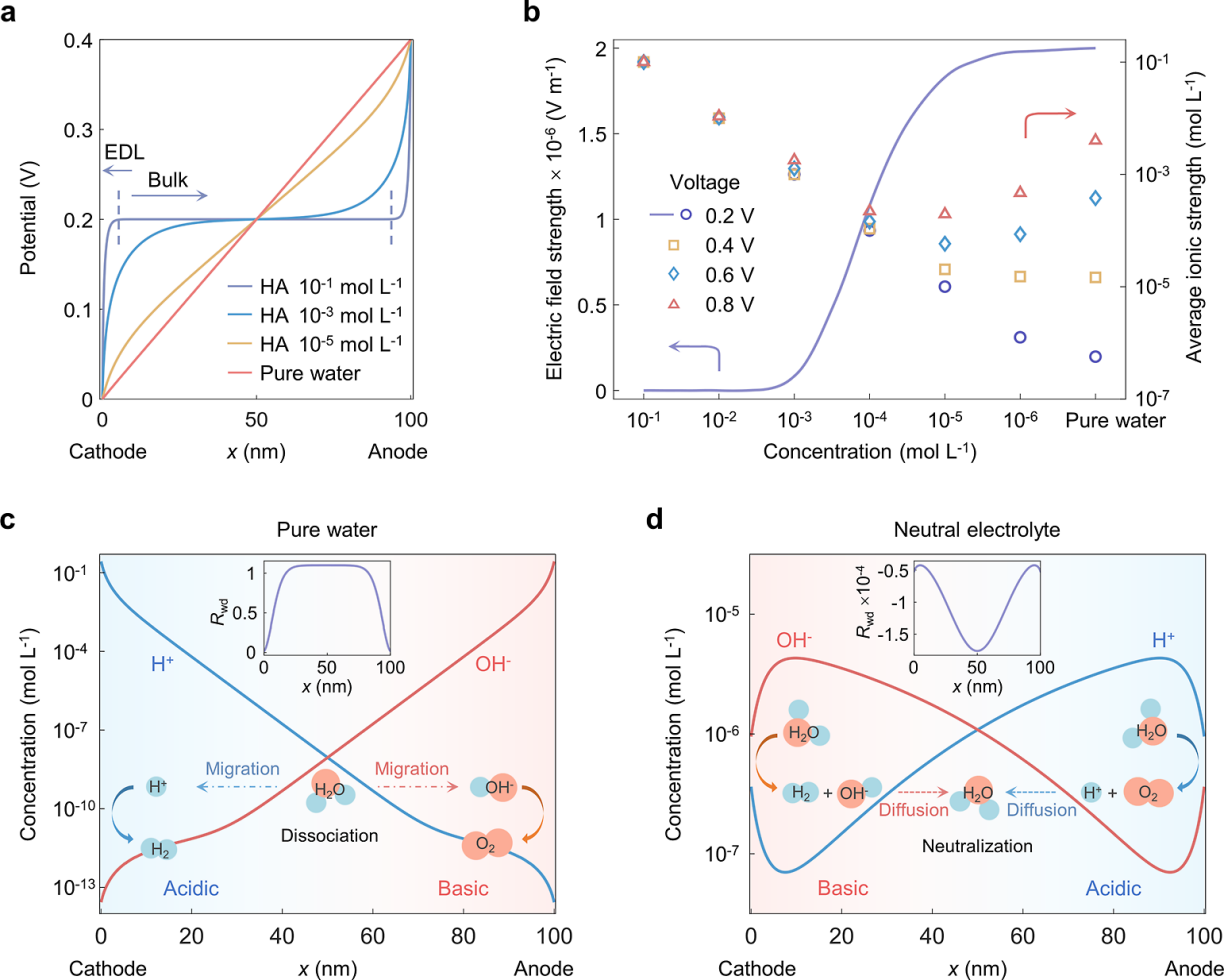
Electric field enhancement, ionic strength augmentation, and favorable acid–base environment in the overlapping EDLs. (a) Distribution of electrostatic potential in electrolyte solution HA (a general denotation for acids with symmetrical ions) at different ion concentrations. The EDLs (demarcated by dashed lines) expand and overlap as the ion concentration decreases. (b) Electric field strength at the center of the electrolyte solution and average ionic strength over the whole electrolyte solution of HA (10^–6^∼10^–1^ mol·L^–1^) and pure water as functions of the applied voltage at an interelectrode distance of 100 nm. The electric field strength intensifies as the concentration of the supporting electrolyte decreases, caused by the higher degree of EDL overlap. The ionic strength first declines as the ion concentration decreases from 0.1 M to 0.1 mM, but then rises with further decreasing the ion concentration and increasing the voltage difference of the two electrodes. (c) An acidic HER and basic OER environment is established during pure water splitting within the overlapping EDLs at a cell voltage of 1.5 V. The inset shows the net rate of water dissociation *R*_wd_ (mol·m^–3^·s^–1^) in the electrolyte solution, where a positive value indicates the occurrence of water dissociation. (d) The concentration distributions of H^+^ and OH^–^ in the electrolysis of a neutral electrolyte reveal a basic HER and acidic OER environment at a cell voltage of 2.5 V. The inset shows a negative *R*_wd_, signifying the occurrence of water neutralization in the electrolyte.

To characterize the overlapping EDLs and understand their effects on pure water splitting, a continuum model is developed. This model, as detailed in Supplementary Note 2, describes ion transport in the overlapping EDLs with water electrolysis reactions occurring at the electrode surface. The water dissociation reaction (*R*_wd_) is incorporated as a source term in the Poisson–Nernst–Planck equation that describes the transport of H^+^ and OH^–^ in water. Additionally, the model considers the transport of cations M^+^ and anions A^–^ of a supporting electrolyte, if present. The kinetics of HER and OER are described using Butler–Volmer equations in both acidic and basic environments. The potential and ion distributions are simulated within an electrode distance of 100 nm, for cases with and without supporting electrolytes (Figure 2).

The characteristic length of EDLs, the Debye length, grows from 1 nm in 10^–1^ M acid to 1 μm in pure water.^38^ As the acid concentration decreases, the region of the bulk solution, signified by a plateau in the distribution of electrostatic potential, gradually diminishes (Figure 2a). In pure water, the potential distribution between cathode and anode is almost linear, indicating an extensive overlap of the EDLs at an interelectrode distance of 100 nm, namely, one-tenth of the Debye length. To quantify the overlap of cathode and anode EDLs, we use the strength of the electric field in the center of the liquid phase as the indicator. We find that it intensifies as the concentration of supporting electrolyte decreases (Figure 2b). The strong pervasive electric field enhances the capability of ion migration, surpassing the rate required to attain the desired current density of water electrolysis by more than 10^3^ times. Conversely, in concentrated solutions with separate EDLs, ion transport relies primarily on diffusion, since migration is weakened by the low electric field in the bulk region of the liquid phase. Moreover, the average ionic strength in the overlapping EDLs increases by a factor of 10^5^ in pure water as the applied voltage is increased from 0.2 to 0.8 V (Figure 2b). The excess ions are produced from the dissociation of water, driven by the necessity to screen the intense electric field in the absence of sufficient supporting electrolytes. This effect gradually diminishes as the ion concentration increases, owing to the weakened strength of the overlapping EDLs. The combined effects of enhanced migration capability and facilitated ionization of water render ion transport between the confronting electrodes in pure water sufficiently close to that in concentrated electrolytes.

An acidic condition for HER and a basic condition for OER are formed *in situ* in pure water splitting (Figure 2c). Driven by the electric field in the overlapping EDLs, H^+^ and OH^–^ accumulate near the cathode and anode, respectively, and react on the respective surfaces. Water molecules dissociate in the electrolyte to replenish the consumed ions. In contrast, an opposite environment is formed in the electrolysis with a neutral supporting electrolyte, as shown in our simulation (Figure 2d) and corroborated in experimental studies.^24,39^ In a neutral electrolyte, water molecules dissociate during both electrode reactions, and OH^–^ and H^+^ are generated at the cathode and anode, respectively. These ions subsequently encounter each other in the electrolyte solution and neutralize back to water. The difference in the bipolar reaction environments between pure water and neutral supporting electrolytes is due to the large disparity in ion migration capability within the overlapping and nonoverlapping EDLs. In the overlapping EDLs, the strong electric field greatly facilitates the migration of H^+^ to the cathode and OH^–^ to the anode, while in the nonoverlapping EDLs, ion transport relies on diffusion driven by concentration gradients (see Supplementary Note 3 and Figure S4 for extended explanations and factors that influence the acid–base environments). In terms of performance, the coexistence of the favorable acidic and basic environments for HER and OER, respectively, maximizes the reaction activity for pure water splitting.

The water dissociation rate in the overlapping EDLs should be significantly accelerated compared to that in bulk water to support a current density as high as 1 A·cm^2^ (Supplementary Note 2). The acceleration mechanisms are a subject of controversy in literature. The second Wien effect is commonly used to describe the influence of the electric field, which is pronounced when the field strength exceeds 10^8^ V·m^–1^.^40^ Also, molecular dynamics simulations have studied the impact of the electric field on OH bonds within and between water molecules,^41,42^ revealing that water can be autoionized by an electric field higher than 3.5 × 10^8^ V·m^–1^. However, the presence of catalysts is considered to be the dominant factor of water dissociation in bipolar membranes.^16,43,44^ Our results indicate that water dissociation can be accelerated without catalysts by an electric field on the magnitude of 10^7^ V·m^–1^.^35,45^ This strength of the electric field does not support water autoionization, yet it can facilitate water dissociation by stretching the OH bond and reorienting water dipoles, resulting in a lower relative permittivity.^46^ Meanwhile, the continuous dissociation is driven by the shift in water balance. At the center plane of the cell, the concentration of H^+^ and OH^–^ both remain below 10^–7^ M (Figure 2c), thereby impeding ion recombination. The low concentration of H^+^ and OH^–^ are attributed to the enhanced ion migration in the overlapping EDLs.

**Figure 3:**
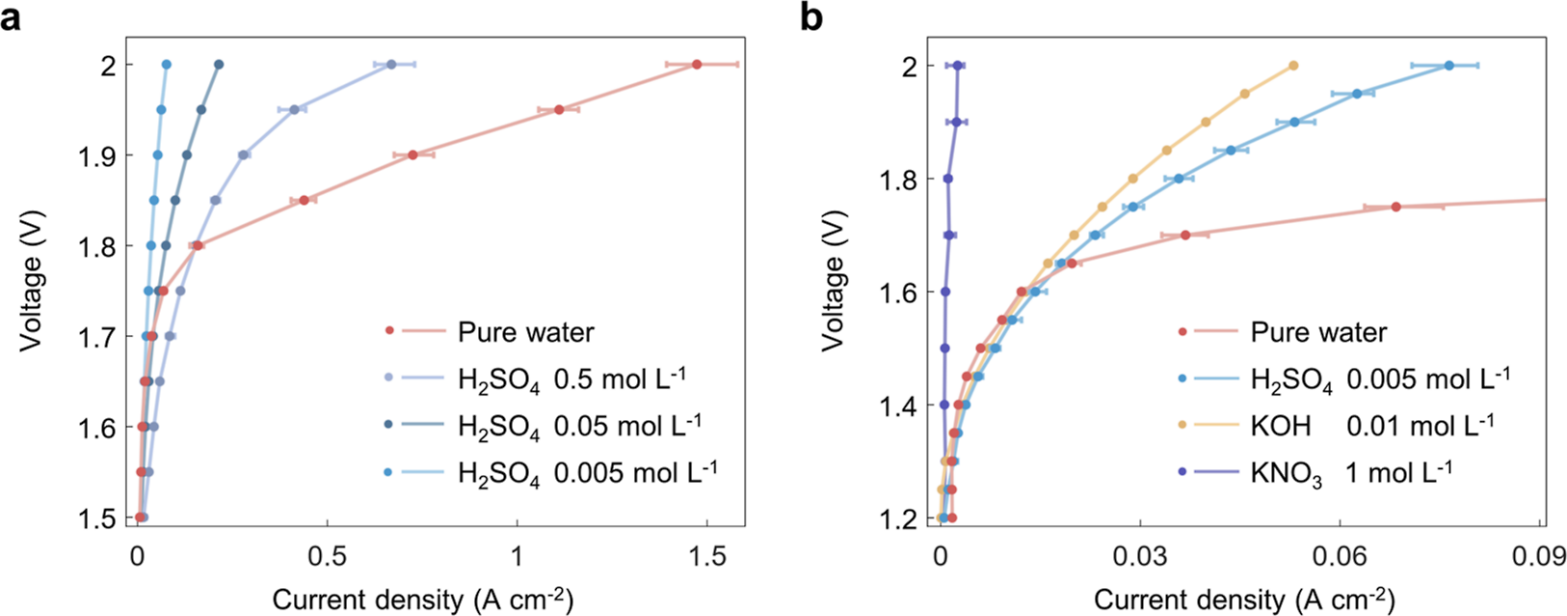
Performance comparison of pure water and supporting electrolytes in the nanofluidic reactor. (a) Pure water splitting outperforms its counterparts with acidic electrolysis. (b) Electrolysis with a KNO_3_ electrolyte shows negligible electrolytic current below 2 V, while H_2_SO_4_, KOH, and pure water deliver currents below 1.4 V, and pure water exhibits superior performance over acid and alkaline electrolytes. The IV curves are measured at 60 °C, 1 m·s^–1^ flow rate, *D* = 100 nm, *L* = 5 μm, and corrected by current efficiency.

Since the overlapping EDLs enhance ion migration and create an acidic HER and basic OER environment, we would expect the electrolytic performance in pure water to be different from that in supporting electrolytes with separate EDLs. Different types and concentrations of electrolytes for water splitting are compared in the nanofluidic reactors (Figure 3). The performance of acidic electrolysis decreases when the electrolyte concentration decreases from 0.5 to 0.005 M (Figure 3a). However, pure water splitting outperforms the case with 0.5 M H_2_SO_4_. The enhanced migration capability and augmented ionic strength in the overlapping EDLs bring the ionic conductivity of pure water close to that of concentrated electrolytes. In addition, the acidic cathode and basic anode environments are beneficial for HER and OER, respectively, leading to a reduction in the overpotential for surface reactions. These two effects of the overlapping EDLs contribute to the superior performance of pure water splitting compared to the cases with supporting electrolytes.

Furthermore, a more pronounced difference is observed when comparing pure water splitting with the case of a neutral electrolyte (Figure 3b). While the neutral electrolyte exhibits no electrolytic current below 2 V, pure water, also originally neutral, initiates electrolysis at voltages below 1.4 V. The high onset potential of the neutral electrolyte is caused by the extra dissociation and neutralization during the basic HER and acidic OER, wasting a thermodynamic potential of 0.83 V.^39,47^ This observation supports the presence of the acidic HER and basic OER during pure water splitting; otherwise, the physics and performance would resemble those of neutral supporting electrolytes.

Notably, the current density of pure water is initially lower than that of 0.5 M H_2_SO_4_ but becomes higher above 1.8 V (Figure 3a). This is attributed to the intensified ionic strength in the overlapping EDLs as the voltage is raised (Figure 2b). Different from conventional electrochemical devices, where the ion concentration and reaction environments are predetermined by added electrolytes, the present pure water splitting reactor forms the electrolyte and the bipolar environment *in situ* above a “voltage threshold”. Subsequently, it exhibits a higher sensitivity to the change of the cell voltage. This suggests a new approach for electrochemical systems with voltage-dependent ionic strength and local reaction environments. This mechanism also brings another advantage in terms of electrolyte purity, as it circumvents issues related to adsorption, poisoning, and other effects of electrolyte species. Thus, it holds the potential to serve as a platform for various fundamental studies on nanoconfined electrocatalysis and electrokinetics.

### Toward High Performance with Low Material Cost

**Figure 4:**
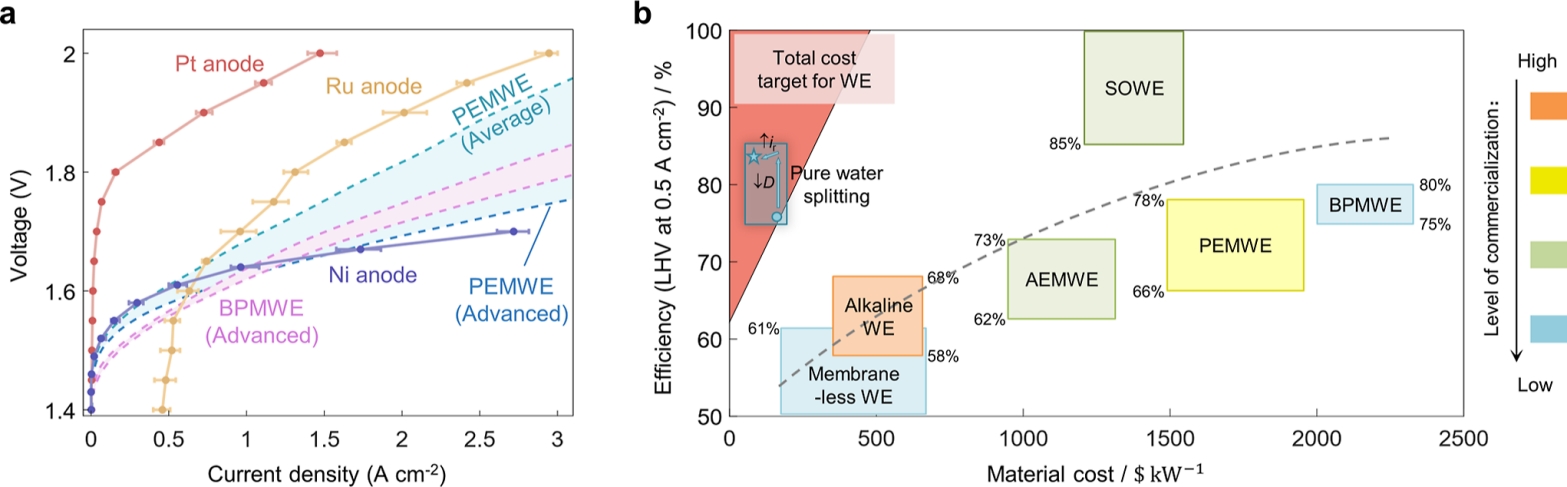
Pure water splitting with a Ni anode and its comparison with other variants of water electrolyzers. (a) Pure water splitting with Ni and Ru as OER catalysts, which have higher activities in alkaline environments, show better performance than that with a Pt anode. The performance of pure water splitting is measured at 60 °C, 1 m·s^–1^ flow rate, *D* = 100 nm, and *L* = 5 μm, with the current efficiency correction. Pure water splitting with suitable basic OER catalysts provides competitive performance in comparison with advanced PEMWE and BPMWE. Meanwhile, it has much lower material costs due to the absence of the membrane and supporting electrolyte, and the potential to use nonprecious metal catalysts. (b) Comparing pure water splitting with the target and status of water electrolyzers. The red triangle represents the requirements of efficiency and material cost to meet the total cost target of water electrolyzers when combined with renewable energy sources at a $0.03 electricity price and a 30% capacity factor. The rectangles represent the status of existing water electrolysis technologies and the dashed line summarizes a trend between the efficiency and the material cost established among these technologies. The efficiencies and the material costs of technologies at higher technology readiness levels (TRLs) are adopted from the literature,^3,4,8,49−53^ while the material costs of the methods at lower TRLs are estimated. WE denotes water electrolyzer, AEMWE is short for anion exchange membrane WE, SOWE denotes solid oxide WE, PEMWE denotes proton exchange membrane WE, and BPMWE denotes bipolar membrane WE. The blue dot represents the performance of pure water splitting in this work, with an efficiency of 76% at 0.5 A·cm^–2^ and lower material cost than alkaline WE and common membrane-less WE. By reducing the electrode distance (*D*) and increasing the rated current density (*i*_r_), it is anticipated that pure water splitting will reach the position of the blue pentagram.

Following the fundamental understanding of the structure of the overlapping EDLs and their effect on the electrolysis performance, we aim to achieve pure water splitting with cheaper materials. Based on the favorable alkaline anode environment in the overlapping EDLs, we replace the Pt anode electrode with ruthenium (Ru) and nickel (Ni) (Figure 4a). The Ni anode enables a significant improvement in pure water splitting performance since Ni possesses a 10^3^-fold higher exchange current density under alkaline conditions than Pt.^5^ This performance enhancement further proves the presence of the basic reaction environment near the anode. The residual current observed with the Ru anode at voltages below 1.4 V results from Ru degradation.^48^ Pure water splitting with the Ni anode can reach 2.8 A·cm^–2^ at 1.7 V, a performance that surpasses the advanced PEMWE and BPMWE.^8,49,50^

Pure water splitting in the overlapping EDLs delivers competitive electrolysis performance with nonprecious materials, which represents a promising path toward low-cost, high-performance water electrolysis. Conventional water electrolyzers typically require supporting electrolytes to enhance the conductivity in the bulk region, such as alkaline water electrolyzers. To further minimize the ohmic resistance, solid electrolytes are employed to reduce the thickness of the bulk. Moreover, achieving higher activity often involves the use of precious metal catalysts as in PEMWE, creating the acid–base environment as in BPMWE, or raising the temperature above 500 °C as in solid oxide water electrolyzers (SOWE). Therefore, those variants are confronted with the well-known challenge of balancing energy efficiency and material cost, rendering it difficult to meet the overall targets (Figure 4b; the cost target is analyzed in Supplementary Note 4 and Figure S5).

In contrast, leveraging the advantages of overlapping EDLs, pure water splitting provides an alternative route to green H_2_ production. Our reactor achieves state-of-the-art performance without the use of membranes, supporting electrolytes, or precious OER catalysts. Moreover, the cathode can also employ earth-abundant materials for the acidic HER.^51^ Efficiency can be further enhanced by reducing the electrode distance (*D*). The current density increases more than 10 times when we decrease *D* from 200 to 100 nm (Figure 1e); additional improvements are expected when *D* is further reduced with a further intensified electric field. In a laminated-type reactor with a 50 nm interelectrode distance, we observe a current density higher than 40 A·cm^–2^ at 2.5 V and 20 °C, which demonstrates the potential for efficiency improvement (Figure S6). Meanwhile, increasing the rated current density (*i*_r_) is advantageous for pure water splitting. In Figure 4b, we choose 0.5 A·cm^–2^ as *i*_r_ for a uniform comparison among methods; however, this underestimates the superiority of pure water splitting. At 0.5 A·cm^–2^, the voltage slightly surpasses the “voltage threshold” for pure water splitting to initiate the ions and environments, as previously discussed (Figure 3a). Increasing *i*_r_ to 3 A·cm^–2^ or higher will lead to a slight decrease in efficiency, but a substantial increase of power density, which will significantly reduce the capacity costs to 30% or less. Consequently, this technique is a promising solution to meet the cost target for H_2_ production.

## Conclusions

We have reported an unconventional electrochemical water-splitting technology that harnesses the overlapping cathode and anode EDLs to split pure water in a nanofluidic reactor. The crossover of H_2_ and O_2_ is effectively mitigated by maintaining a sufficient convective flow in the nanochannel. The current efficiency of the reactor has been quantified using a pair of detector electrodes downstream of the channel. The overlap of cathode and anode EDLs gives rise to a pervasive electric field on the magnitude of 10^7^ V·m^-1^, facilitating water dissociation and ion migration. A 5-order increase in the ion concentration is obtained in pure water, enabling an ionic conductivity comparable to that of a normally concentrated electrolyte. Furthermore, an acidic HER and basic OER environment are formed *in situ* in pure water, reducing the kinetic overpotential for both reactions and enabling the usage of nonprecious metal catalysts like Ni. Leveraging the synergy of these factors, a Ni-based pure water reactor can achieve a performance of 2.8 A·cm^–2^ at 1.7 V with no supporting electrolytes. This study suggests a promising solution for green H_2_ production with high energy efficiency at low material cost.

While our study shows promising results, scalability emerges as a critical consideration for raising the TRL of this technology. In our cell design, we have considered the issue of scaling up by incorporating a flow field into the reactor, allowing for integration and product output. However, extending the electrode area to a practical level while maintaining the nanoscale electrode distance presents a significant challenge. Addressing this challenge requires efforts toward designing extendable and repeatable electrode units, integrating multiple electrodes, and interconnecting multiple reactors. Lastly, we emphasize that, along with the engineering challenges, this work also unveils several open scientific questions for future research, including the atomistic mechanism of facilitated water splitting in the overlapping EDLs, experimental probing of the local reaction conditions at the cathode and anode, as well as the dynamic behaviors of this device.

## Supporting Information Available

Detailed fabrication processes and experimental procedures, calibration of the H_2_ detector, modeling of overlapping EDLs, discussion of factors that influence the acid–base environments, cost target analysis for water electrolyzers, and performance of pure water splitting using a laminated-type reactor with 50 nm interelectrode distance (PDF)
