# Supplementary material for: Pure Water Splitting Driven by Overlapping Electric Double Layers
Source: J Am Chem Soc. 2024 Jul 10;146(29):19720–7. doi: 10.1021/jacs.4c01070 (PMC11273347; doi:10.1021/jacs.4c01070)
Supplement: Supplementary file 1 — ja4c01070_si_001.pdf [file ja4c01070_si_001.pdf]

Supporting Information for

Pure Water Splitting Driven by Overlapping Electric Double Layers

Haosen Xu,[1,2] Jianbo Zhang, [1,*] Michael Eikerling,[2,3] Jun Huang,[2,4,*]

[1] School of Vehicle and Mobility, State Key Laboratory of Intelligent Green Vehicle and Mobility, Tsinghua University, 100084, Beijing, China

[2] IEK-13, Institute of Energy and Climate Research, Forschungszentrum Jülich GmbH, 52425, Jülich, Germany

[3] Chair of Theory and Computation of Energy Materials, Faculty of Georesources and Materials Engineering, RWTH Aachen University, 52062, Aachen, Germany

[4] Theory of Electrocatalytic Interfaces, Faculty of Georesources and Materials Engineering, RWTH Aachen University, 52062, Aachen, Germany

[*] Corresponding author, e-mail: ju.huang@fz-juelich.de, jbzhang@tsinghua.edu.cn

This file includes:

Methods

Supplementary Note 1 to 4

Figure S1 to S6

Table S1

Supplementary References

Methods

Fabrication of nanofluidic reactor

The nanofluidic reactor is composed of two pairs of confronting electrodes. Each pair comprises two electrodes disposed on the top and bottom sides of a nanochannel, which is manufactured by etching off a sacrifice layer between the electrodes. Additionally, microchannels are fabricated on the chip and connected to the nanochannel via conduits. A cube of polydimethylsiloxane (PDMS) with inlet and outlet holes is bonded with the chip to seal the upper ends of the microchannels. The fabrication employs photolithography and semiconductor techniques, and the process includes deposition, etching, and bonding steps (Figure S1).

On a Si wafer with a SiO_2 layer of 500 nm thickness, notches with the pattern of bottom electrodes are etched by reactive ion etching (RIE) to a depth of 50 nm. Then, Cr adhesion layers of 5 nm and Pt bottom electrodes of 45 nm are deposited by electron-beam evaporation (EBE) in sequence to flatten the notches. This sequential process of etching and deposition prevents the bottom electrodes from protruding into the nanochannel, thus maintaining the straightness of the nanochannel. Subsequently, a sacrifice layer of Ti is deposited by EBE to separate the bottom and top electrodes. The thickness of the sacrifice layer is the key parameter that regulates the distance between the confronting electrodes. We use different materials for the top electrodes, including Pt, Ru, and Ni, and cover them with a Cr adhesion layer. Upon the completion of the deposition step, the electrode region is passivated by a 2.5 μm SiO_2 layer using plasma-enhanced chemical vapor deposition (PECVD).

The microchannels with a depth 2 μm and the conduits straight down to the sacrifice layer are etched in the SiO_2 passivation layer using RIE. It is necessary to coordinate the scale transition through the conduits to the nanochannel. Our preliminary experiments for reactor designs revealed that water cannot be purged into the nanochannel if the microchannels are directly etched down to the nanochannel without conduits. Following the fabrication of microchannels and conduits, we select one chip located in each corner of the wafer and cut them open using the focused ion beam (FIB), to confirm the structure of the electrodes and the thickness of the sacrifice layer (Figure S1b). The rest of the chips are immersed in ammonia (10%) for more than 24 hours to etch away the sacrifice layer, thereby forming the nanochannel. The wet etching process can be monitored optically and electrically by measuring the resistance between the confronting electrodes.

After the etching step, the chip is bonded with a PDMS cube of 3 mm thickness to seal the upper boundaries of the microchannels. The bonding strength is vital, as the bonding interfaces must withstand the high pressure caused by the nanoscale convection. The interfaces of the chip and PDMS are treated with O_2 plasma (Alpha Plasma Q150) at 30°C, 60 Pa chamber pressure, 200 sccm O_2 flow, and 200 W plasma power. We find that subjecting the chip to two rounds of plasma treatment, with an intermediate ethanol wash, significantly enhances the bonding strength. After the plasma treatments, we adhere the PDMS onto the chip and heat them at 120°C for more than 2 hours. Then, the nanofluidic reactor is prepared (Figure S1c). Holes are punched at the nodes (200 μm height) reserved within the PDMS to work as inlets and outlets for electrolytes.

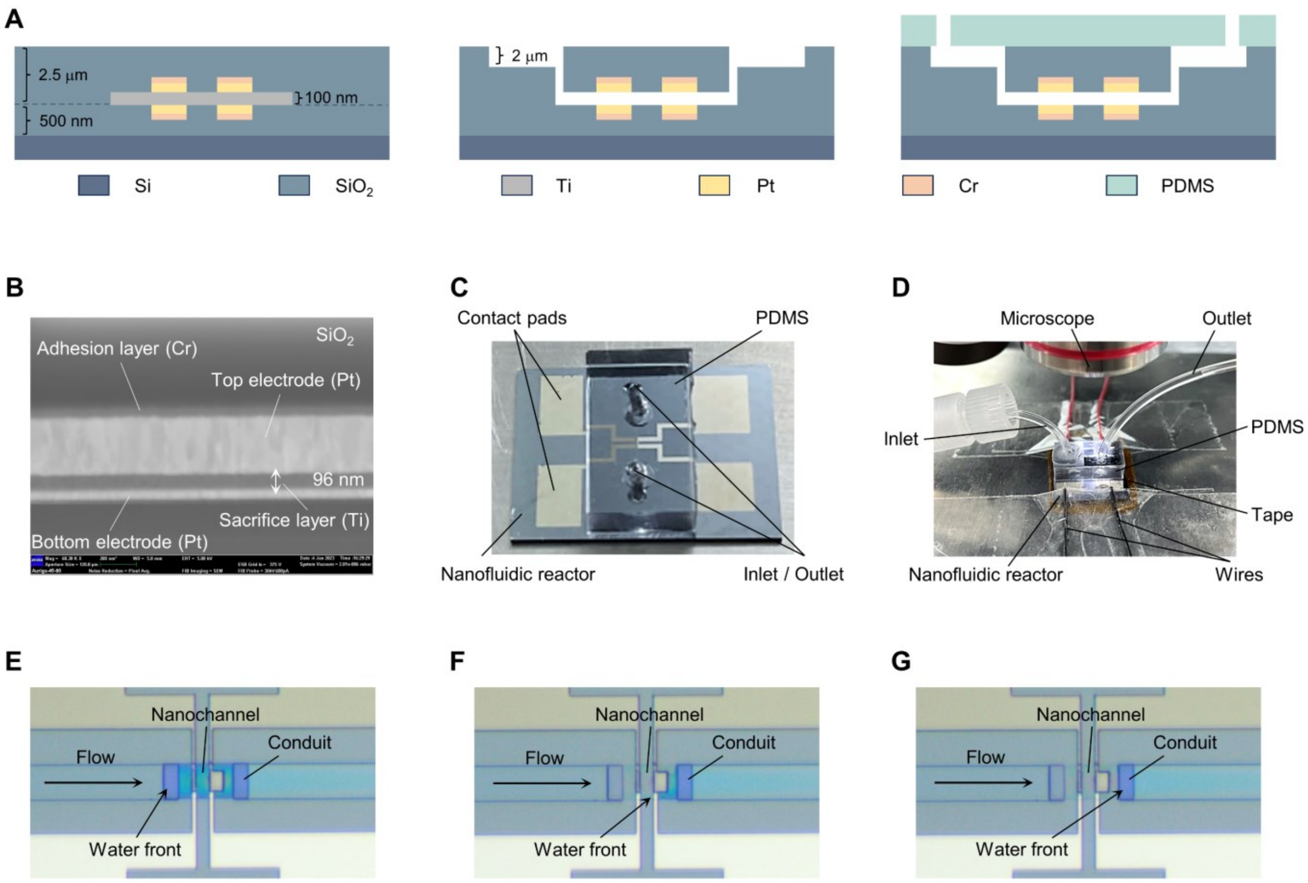

Figure S1. Fabrication process of the nanofluidic reactor and the experimental setup. (**a**) Schematic of the three-step fabrication process for the nanofluidic reactor: deposition of Pt electrodes, Cr adhesion layers, Ti sacrifice layer, and SiO_2 passivation layer (left); etching of the passivation layer to form microchannels and conduits, and wet etching of the sacrifice layer to form the nanochannel (middle); bonding with PDMS to seal the upper ends of the microchannels. (**b**) Scanning electron microscope (SEM) image of the cross-section of the electrodes, cut open by the FIB. The thickness of the sacrifice layer is measured to confirm the distance between the confronting electrodes. (**c**) The overall image of the nanofluidic reactor after bonding with PDMS. Contact pads on the wafer serve as connection points for external wires. The inlet and outlet of water are punched out in the PDMS. (**d**) Water and electron connections of the nanofluidic reactor. (**e-g**) Observation of the water purging process in the nanochannel. Water is propelled into the nanochannel through the upstream conduit and flows out from the downstream conduit.

<u>Electrolyte injection and observation</u>

The nanofluidic reactor is connected to a syringe pump (Chemxy Fusion 100X) to inject electrolytes into the nanochannel (Figure S1d). The tubing is inserted into the inlet hole of the PDMS and glued at the interface (3M AC78 silicone pretreatment agent and 3M CA40H quick-drying glue). Prior to be injected into the reactor, the ultra-pure water is N_2 depleted to prevent the dissolution of CO_2, and the solutions with added electrolytes are titrated to confirm the

concentration.

The injection process is observed through the microscope, capturing the water flow through three stages: flowing in the inlet microchannel (Figure S1e), being injected into the inlet conduit and nanochannel (Figure S1f), and flowing out to the outlet conduit (Figure S1g). Then, the flow rate is maintained at 0.5 $m \cdot s^{-1}$ for 12 h to ensure the stability of the reactor structure and complete immersion of the electrodes. In the subsequent electrochemical tests, any alteration in flow rate needs to wait for more than 30 min and is monitored by a force sensor at the bottom of the syringe to confirm the flow stability.

Electrochemical methods

Electrochemical tests are conducted in a temperature-controlled chamber (ESPEC Co., Ltd) with two nested Faraday cages, and managed using an electrochemical workstation (Metrohm Autolab PGSTAT302N). A temperature-controlled heating plate is positioned beneath the chip to warm up the chip from the original 20°C to 60°C. Wires are attached to the contact pads on the chip by silver conductive paint. The top electrodes function as the cathode and the bottom electrodes serve as the anode. The nanofluidic reactor is initially activated by the procedure of a linear sweep voltammetry (LSV) from 0 V to 2 V followed by a cyclic voltammetry (CV) from -1 V to 1 V to refresh the electrodes. The activation process continues until the observed performance shift between two consecutive LSVs remains within 5 mV at the same current. Then, the current is stabilized for 10 min under each voltage from 1.2 V to 2 V with 50 mV intervals to characterize the current-voltage (IV) curves of water electrolysis.

Supplementary Notes

Supplementary Note 1: Calibration of H_2 detector

The detector electrodes are embedded downstream of the nanochannel to quantify the current efficiency of pure water splitting occurring in the reactor electrodes. However, two conditions complicate the detection of H_2: one is that H_2 is dissolved in ultra-pure water; the other is the mixing of O_2 in H_2. Pure water, with an ionic strength of 10^{-7} M, results in high ohmic resistance, decreasing the response current and preventing the establishment of the reference electrode. To address this problem, the detector electrodes are designed in the confronting structure with a nanometer distance to reduce the ohmic resistance between the electrodes. Meanwhile, the overlapping electric double layers (EDLs) of the detector electrodes can elevate ionic strength and accelerate ion migration in the electrolyte.

To manage the issue of the mixing of O_2 in H_2, the characteristic voltage for H_2 needs to be determined based on the different properties between dissolved H_2 and O_2. Pure water with different dissolved gases is prepared by bubbling and boiling in a Monteggia bottle. A magnetic stirrer is positioned at the bottom of the bottle and rotates constantly. Pure water is boiled to remove the original dissolved gases, and then cooled down to room temperature, while the specific gas to be dissolved is continuously bubbled into the water. H_2, O_2, and N_2 are individually dissolved in pure water. The concentration of H_2 is determined using a portable dissolved H_2 sensor (Trustlex ENH-2000).

The linear sweep voltammetry (LSV) of the detector reveals distinctive behavior of the pure water dissolved with H_2, O_2, and N_2, individually (Figure S2). The current of dissolved H_2 exhibits an initial rise upon the application of voltage and saturates after reaching 0.2 V. At higher voltages, the current continues to show a slight increase, owing to the increase of ionic strength within the overlapping EDLs. In contrast, the signal of O_2 can be identified only when the voltage surpasses 0.5 V. This difference stems from the massive discrepancy in the reaction kinetics of H_2 and O_2. In the confronting detector electrodes, the cathode occurs the reduction reaction of H_2 or O_2, while the anode occurs the evolution reaction of the gases. The voltage difference represents the sum of the overpotential of the reactions on the two electrodes. The rapid kinetics of the reduction and evolution reactions of H_2, exceeding 10^5-fold that of O_2, accounts for the significantly lower overpotential. Thus, the reactions of H_2 can occur below 0.2 V, while the current of O_2 can only be detected above 0.5 V. For the N_2-depleted pure water, lacking any dissolved gas reactant, the observed current solely represents the oxidation of Pt, evident at approximately 0.9 V. Consequently, the range of the detection voltage for H_2 is determined between 0.2 V to 0.5 V, with current for H_2 reaching saturation while remaining insensitive to O_2. Therefore, we select 0.4 V as the detection voltage for H_2. Then, we calibrate the detector and find that the current is linearly proportional to the dissolved H_2 concentration when the system reaches the current-saturated state for H_2 under 0.4 V (Figure S3).

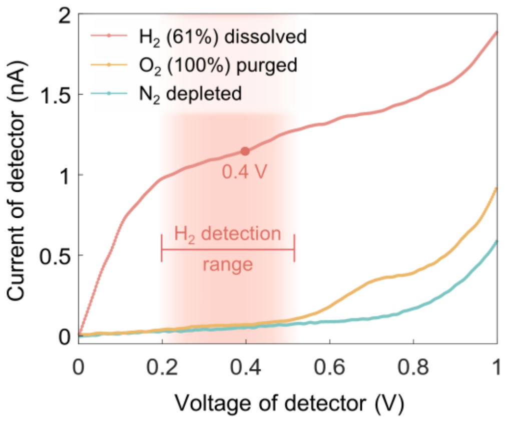

Figure S2. Characteristic voltage for H_2 detection. LSVs of the detector electrodes, ranging from 0 V to 1 V, exhibit distinct characteristics among pure water with H_2, O_2, and N_2 dissolved individually. The current of the detector with dissolved H_2 rises rapidly from 0 V to 0.2 V and maintains a consistently high level thereafter. In contrast, the signal of O_2 only becomes recognizable above 0.5 V. The red range of voltage is capable of H_2 detection with adequate current response for H_2 while remaining insensitive to O_2. Thus, 0.4 V is selected as the optimal characteristic voltage of H_2. The LSVs are measured at 20°C, using a scan rate of 20 $mV\cdot s^{-1}$, in a detector with 200 nm electrode distance and 20 μm electrode width.

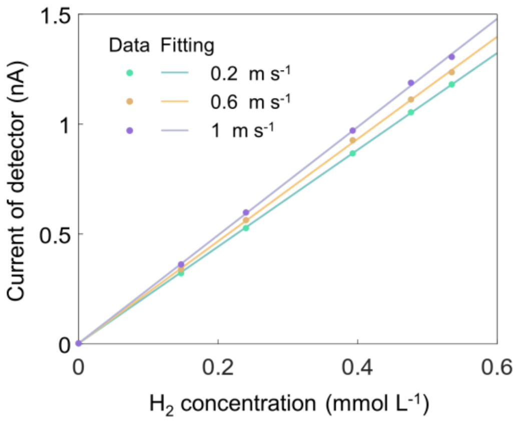

Figure S3. Relationship between detector signal and H_2 concentration. The current of the detector is measured in pure water with various concentrations of dissolved H_2 and flow rates. The experiments are conducted at 20°C in the detector with 200 nm electrode distance and 20 μm electrode width.

After the calibration of the detector, we can calculate the current efficiency of pure water splitting. We apply 2 V to the reactor and 0.4 V to the detector under various flow rates u, and record the current of the reactor i_{re} and the detector i_{de}. The concentration of H_2 that flow out of the reactor $c_{\mathrm{H_2}}$ can be obtained using the fitted relationship in Figure S3, $c_{\mathrm{H_2}} = f(i_{\mathrm{de}})$.Then,

the current efficiency can be calculated,

$$\eta_{\mathrm{c}} = \frac{2F \cdot f(i_{\mathrm{de}}) \cdot uWD}{i_{\mathrm{re}}}, \tag{1}$$

where F is the Faraday's constant, W and D denote the width and depth of the nanochannel. The term uWD is the volumetric flow rate of pure water.

Supplementary Note 2: Modeling of overlapping EDLs

A physical model is developed to capture the properties of the overlapping cathode and anode EDLs. In the classic model of EDLs, potential distribution and ion transport are described by the Poisson-Nernst-Planck theory[1]. However, water dissociation reaction serves as a non-negligible source of ions in ultra-dilute solutions under strong electric fields. Thus, we add the water dissociation as a source term in the ion conservation equations for H^+ and OH^-,

$$\frac{\partial c_{\mathrm{H^+,OH^-}}}{\partial t} = \frac{\partial}{\partial x}\left(D\frac{\partial c_{\mathrm{H^+,OH^-}}}{\partial x} + \frac{zF}{RT}Dc_{\mathrm{H^+,OH^-}}\frac{\partial \phi_{\mathrm{e}}}{\partial x}\right) + R_{\mathrm{wd}}, \tag{2}$$

where $c_{\mathrm{H^+,OH^-}}$ represents the concentration of H^+ or OH^-, D is the diffusion coefficient of the ions, z is the charge number, R is the gas constant, T is the temperature, ϕ_{e} is the potential in the electrolyte, and t and x refer to the time and position, respectively. R_{wd} stands for the source term of the water dissociation reaction,

$$R_{\mathrm{wd}} = k_{\mathrm{d}}c_{\mathrm{H_2O}} - k_{\mathrm{n}}c_{\mathrm{H^+}}c_{\mathrm{OH^-}}, \tag{3}$$

where k_{d} and k_{n} refer to the reaction rate constants of dissociation and neutralization, respectively. $c_{\mathrm{H_2O}}$ is the concentration of water and is considered as constant.

For ions from added electrolytes, denoted by M^+ and A^-, the ion conservation equation is given as,

$$\frac{\partial c_{\mathrm{M^+,A^-}}}{\partial t} = \frac{\partial}{\partial x}\left(D\frac{\partial c_{\mathrm{M^+,A^-}}}{\partial x} + \frac{zF}{RT}Dc_{\mathrm{M^+,A^-}}\frac{\partial \phi_{\mathrm{e}}}{\partial x}\right). \tag{4}$$

The potential distribution in the EDLs follows the Poisson equation,

$$\frac{\partial}{\partial x}\left(\varepsilon_{\mathrm{r}}\varepsilon_0\frac{\partial \phi_{\mathrm{e}}}{\partial x}\right) = -F\sum_i z_i c_i,$$

where ε_{r} is the relative permittivity of the electrolyte solution, ε_0 is the vacuum permittivity, i represents the ion species in the electrolyte.

For boundary conditions, the classic EDL models commonly account for only one electrode. These conditions involve setting the electrode potential, along with the potential and ion concentration in the bulk of the electrolyte. However, in the overlapping EDLs, the bulk region is excluded from the system. The potential and ion concentration within the electrolyte cannot be predetermined, due to the non-equilibrium state of the system during the occurrence of pure water splitting. Thus, we take the potential of the two electrodes as the boundary conditions,

$$\phi_{\mathrm{s,c}} = 0, \phi_{\mathrm{s,a}} = V, \tag{5}$$

where $\phi_{\mathrm{s,c}}$ and $\phi_{\mathrm{s,a}}$ represent the electrode potential of the cathode and anode, respectively. V is the potential difference applied to the reactor.

The reactions on the electrodes are described by the Bulter-Volmer equation. We consider the H_2 evolution reaction (HER) and O_2 evolution reaction (OER) in both acidic and basic environments,

$$i_{\mathrm{HER,acid}} = i_{\mathrm{0,HER,acid}}\left(\frac{c_{\mathrm{H^+}}}{c_{\mathrm{H^+}}^{\mathrm{ref}}}\exp\left(-\frac{\alpha_{\mathrm{HER,acid}}F\eta}{RT}\right) - \exp\left(\frac{(1-\alpha_{\mathrm{HER,acid}})F\eta}{RT}\right)\right), \tag{6}$$

$$i_{\mathrm{HER,base}} = i_{0,\mathrm{HER,base}}\left(\exp\left(-\frac{\alpha_{\mathrm{HER,base}}F\eta}{RT}\right) - \frac{c_{\mathrm{OH^-}}}{c_{\mathrm{OH^-}}^{\mathrm{ref}}}\exp\left(\frac{\left(1-\alpha_{\mathrm{HER,base}}\right)F\eta}{RT}\right)\right), \quad (7)$$

$$i_{\mathrm{OER,acid}} = i_{0,\mathrm{OER,acid}}\left(\exp\left(\frac{\alpha_{\mathrm{OER,acid}}F\eta}{RT}\right) - \frac{c_{\mathrm{H^+}}}{c_{\mathrm{H^+}}^{\mathrm{ref}}}\exp\left(-\frac{\left(1-\alpha_{\mathrm{OER,acid}}\right)F\eta}{RT}\right)\right), \quad (8)$$

$$i_{\mathrm{OER,base}} = i_{0,\mathrm{OER,base}}\left(\frac{c_{\mathrm{OH^-}}}{c_{\mathrm{OH^-}}^{\mathrm{ref}}}\exp\left(\frac{\alpha_{\mathrm{OER,base}}F\eta}{RT}\right) - \exp\left(-\frac{\left(1-\alpha_{\mathrm{OER,base}}\right)F\eta}{RT}\right)\right). \quad (9)$$

Here, equations 6 to 9 depict the reaction kinetics of acid HER, basic HER, acid OER, and basic OER, respectively. i_0 is the exchange current density corresponding to the reference concentration c^{ref}, and α is the transfer coefficients calculated from the experimentally measured Tafel slop of these reactions. Overpotential η is denoted as the potential difference between the electrode and electrolyte near the electrode surface, expressed by $\phi_s - \phi_e - E^{\theta}$, with E^{θ} being the thermodynamic potential of electrode reactions. The parameters of this model are listed in Table S1.

This model does not consider the detailed mechanisms of reactions with adsorbed intermediates, and neglects the impact of the electric field on dielectric permittivity and water dissociation rate. For instance, the water dissociation rate is certainly enhanced by the strong electric field in pure water splitting. If we use the unaccelerated dissociation rate constants (k_{d}=2×10^{-5} s^{-1}) and the constant water concentration ($c_{\mathrm{H_2O}}$=55.6 M), the highest water dissociation rate, neglecting the counter-reaction of neutralization, can only support a current density of 10^{-6} A·cm^{-2}. This contrasts with our experimental data on pure water splitting, which exhibits a current density of up to 3 A·cm^{-2}. The water dissociation is significantly accelerated by the strong electric field, but this cannot be quantitatively depicted by the theory of the second Wien effect, which is only pronounced when the field strength is 10 times higher than that in our overlapping EDLs[2,3]. The possible mechanisms include the alternation of the H-bond network that results in a lower relative permittivity, and the stretching of the OH bond that facilitates bond breaking.

Supplementary Note 3: Formation of acid-base environment

Acid-base environments are formed in the electrolysis of both pure water and neutral electrolytes. An acidic cathode and basic anode environment is generated in the overlapping EDLs during pure water splitting, while the opposite environment is formed in neutral electrolytes. This phenomenon, as elucidated by the model simulations, is corroborated by the disparities in onset potential of pure water and neutral electrolyte observed in our experiments (Fig. 3b). This reversal of the environment is determined by the relative magnitudes of ion migration capability and required flux. In the non-overlapping EDLs, the strength of the electric field diminishes rapidly from the electrode toward the bulk of the electrolyte. As a result, only in the region near the electrode, the migration capability exceeds the requirement of the total flux, while diffusion is necessary to support the flux in the region further away from the electrode. This leads to a transition of direction in the flux of diffusion and the formation of peak concentration of ions (Figure S4a). The reliance on diffusion in the bulk promotes the accumulation of generated OH^- at the cathode, thereby establishing a basic cathode, and similarly, an acidic anode within neutral electrolytes. On the contrary, the migration capability in the overlapping EDLs is more than 10^3 times higher than the required flux in the typical current density of pure water splitting, leading to the formation of the acidic cathode and basic anode environment. In this environment, the reactants are transformed from water molecules to H^+ at the cathode and OH^- at the anode.

The acidic cathode and basic anode environment is not limited to the context of pure water, but is a distinctive feature of the overlapping EDLs. As the EDLs are gradually brought to overlap with a decrease in the concentration of the neutral electrolyte, the ion peak gradually shifts towards the direction of the electrolyte, concomitant with a decline in basicity (Figure S4a). More effectively, by reducing the electrode distance to 30 nm, the convergence of ion peaks near the two electrodes occurs, leaving only the portion where the migration capability surpasses the required flux (Figure S4b). This alteration induces a shift in the environment of the neutral electrolyte from its original state to an acidic cathode and basic anode configuration. However, the degree of acidity and basicity in the neutral electrolyte are much smaller than those present in the pure water, because the supporting electrolyte apportioned the influence of the electric field.

Notably, the model results demonstrate the features of the overlapping and non-overlapping EDLs in general cases. The neutral electrolytes in the non-overlapping EDLs can also form an acidic cathode and basic anode environment when no current or faint current is applied.

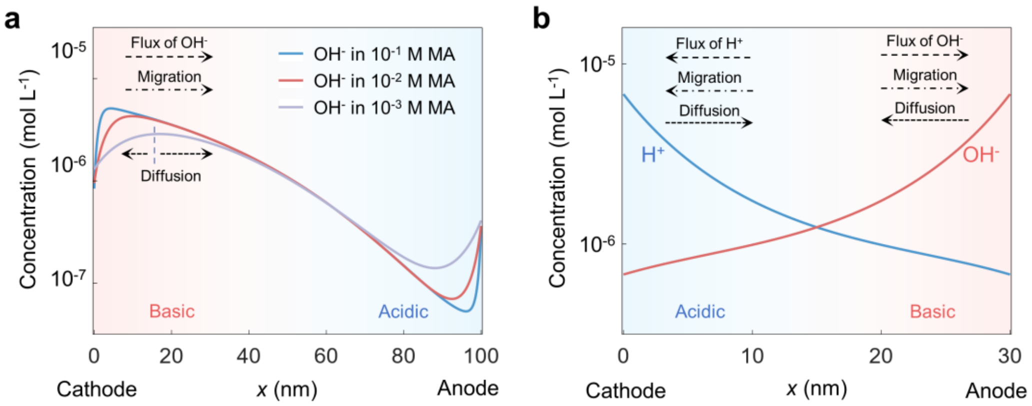

Figure S4. Influence of electrolyte concentration and electrode distance on the acid-base environment. (**a**) A basic cathode and acidic anode environments is formed in MA (a general denotation for neutral electrolytes with symmetrical ions M^+ and A^-) within a 100 nm electrode distance. The distribution of OH^- exhibits a peak in proximity to the cathode. As the concentration of MA decreases, the peak position gradually moves away from the electrode, and its basicity is reduced. The distribution of H^+ follows the same shape and trend as that of OH^-, but mirrored. (**b**) Reducing the electrode distance to 30 nm leads to the reverse of acidity and basicity in 10^{-3} M MA. The ion distributions are simulated under the same current density of 0.01 $A{\cdot}cm^{-2}$.

Supplementary Note 4: Target analysis for water electrolyzers

The integration of solar and wind power sources imposes new requirements on water electrolyzers, differing from those of stable grid supply. This transition can potentially halve electricity costs from current 0.067 $\$\cdot kWh^{-1}$ to 0.03 $\$\cdot kWh^{-1}$. However, it also lowers the capacity factor of electrolyzers to 20% ~ 40%, owing to the fluctuations of renewable energy[4]. That means only around 30% of H_2 can be generated in comparison to full capacity operation with a stable grid power supply. This situation leads to an increase of the capital cost within the total cost of ownership (TCO), while the significance of energy efficiency remains paramount. In the renewable future, the TCO of H_2 produced by water electrolysis should be competitive to serve as a substitute for steam methane reforming (SMR), which relies on natural gas. The cost of H_2 from SMR currently stands at 1.59 $\$\cdot kg^{-1}$, which sets a rigorous benchmark for water electrolyzers[4].

The estimation of the full life-cycle total cost of ownership (TCO) for water electrolysis involves the consideration of both capacity expenditure (Capex) and operating costs (Opex), with the major contributors being material costs and electricity costs, respectively. The capacity cost in each kilogram H_2 is given by,

$$Capex = \frac{p_{\mathrm{mat}} \cdot i_{\mathrm{r}} E^{\theta}}{\eta} \cdot \frac{2F}{f_{\mathrm{ca}} \cdot i_{\mathrm{r}} M_{\mathrm{H_2}} t_{\mathrm{lc}}}, \tag{10}$$

where p_{mat} is the material cost, η is the energy efficiency at the rated current density $i_{\mathrm{r}} = 0.5\ \mathrm{A \cdot cm^{-2}}$, $E^{\theta} = 1.23\ \mathrm{V}$ is the thermodynamic potential of water electrolysis, f_{ca} is the capacity factor, $M_{\mathrm{H_2}} = 2\ \mathrm{g \cdot mol^{-1}}$ is the molar mass of H_2, and t_{lc} is the full life-cycle time of water electrolyzers, chosen as 10 years. The first term on the right side of the equation refers to the total material cost of the electrolyzer, while the second term represents the reciprocal of the total amount of produced H_2. The operating cost is given by,

$$Opex = p_{\mathrm{ele}} \cdot \frac{2FE^{\theta}}{\eta M_{\mathrm{H_2}}}, \tag{11}$$

where p_{ele} is the electricity price. The second term on the right side represents the energy expended to produce 1 kg of H_2.

To achieve a TCO lower than 1.59 $\$\cdot kg^{-1}$, the energy efficiency and material cost of water electrolyzers are estimated for scenarios considering both 100% and 30% capacity factors (Figure S5). As the capacity factor decreases from 100% to 30% due to the inherent fluctuations in renewable energy sources, the requirements to achieve the specified target become notably more stringent, particularly concerning the material cost, while the energy efficiency remains important. To meet the target, water electrolyzers are demanded to achieve the current density of 0.5 $A\cdot cm^{-2}$ with a voltage of 1.55 V, while simultaneously maintaining the material cost under 250 $\$\cdot kW^{-1}$.

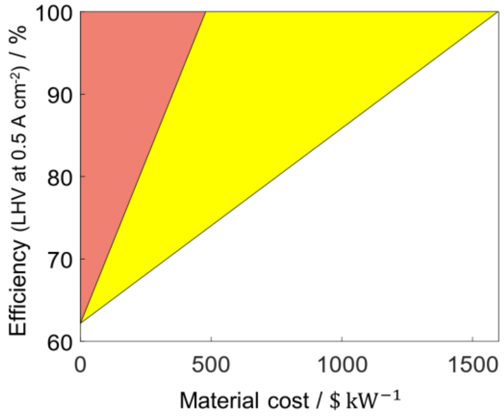

Figure S5. Total cost analysis of water electrolyzers to achieve the cost target. In the context of an electricity price of 0.03 $·kWh^{-1} and a capacity factor of 30%, the energy efficiency, represented by the low heating value (LHV) in comparison to the standard value of 1.23 V, as well as the material cost, should remain within the region of the red triangle. In contrast, the yellow triangle represents the cost target in the scenario where the electricity price remains at 0.03 $·kWh^{-1}, while the capacity factor is at 100%.

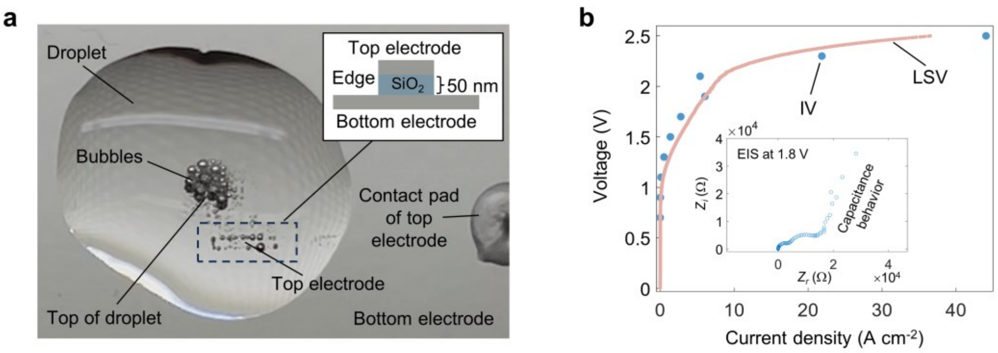

Figure S6. Pure water splitting using a laminated-type reactor with 50 nm inter-electrode distance. (**a**) Image of the top view of the laminated-type reactor during pure water splitting. The insert shows the cross-section side view of the reactor structure. The top and bottom electrodes are made of Pt with 50 nm thickness, separated by a SiO_2 layer of 50 nm thickness. A droplet of pure water is dripped onto the reactor and a voltage of 2 V is applied through contact pads and wires. Bubbles evolute at the edge region of the laminated electrodes, where the overlapping EDLs are located, and move upwards and accumulate at the top of the droplet. We did not utilize the nanofluidic confronting electrodes to assess the performance of the 50 nm electrode distance due to the extremely high pressure required to inject water into the nanochannel of 50 nm depth. (**b**) IV and LSV of pure water splitting on the laminated-type reactor at 20°C. The temperature is not raised to 60°C because the water droplet will evaporate very quickly. Even under room temperature, pure water splitting still exhibits an extremely high current density up to 45 $A \cdot cm^{-2}$ at 2.5 V. The current density is defined as the ratio of total current to the edge area of the top electrode. The performance exhibits dual-slope behavior above 1 V. However, the first slope observed between 1 V to 2 V is attributed likely to the electrical conduction of the intermediate layer surface, rather than water splitting. This analysis is further confirmed by the electrochemical impedance spectroscopy (EIS) conducted at 1.8 V, which reveals a capacitance behavior in the low-frequency region, as depicted in the inset. The electrical conduction arises from the adsorption of ions onto the surface of the silicon-based intermediate layer following the addition of water, and the charged surfaces were shown to be conductive.[5,6] Hence, the intermediate layer should be avoided, as it causes short-circuiting. This issue is effectively circumvented in our nanofluidic reactor. Although the current of short circuit and side reactions caused by the crossover of H_2 and O_2 is included in the total current (the side reaction current cannot exceed half of the total current), and the reaction area also includes the region with non-overlapping EDLs (this region contributes the minor part of the current), this pilot experiment still demonstrates the potential of performance improving with the decrease of the electrode distance.

Table S1. Parameter in the model of overlapping EDLs.

Parameters and description	Value	Unit	Ref.
k_{d}, rate constant of water dissociation reaction	2×10^{-5}	s^{-1}	7
k_{n}, rate constant of water neutralization reaction	1.5×10^{8}	$m^3\ mol^{-1}\ s^{-1}$	7
$c_{\mathrm{H_2O}}$, concentration of water in aqueous solutions	55.6	$mol\ L^{-1}$	7
D, diffusion coefficient of ions	9.1×10^{-9}	$m^2\ s^{-1}$	8
ε_{r}, relative permittivity of water	78.5	-	1
$i_{0,\mathrm{HER,acid}}$, exchange current density of HER in acidic media on Pt	4.2×10^{-4}	$A\ cm^{-2}$	9,10
$i_{0,\mathrm{HER,base}}$, exchange current density of HER in alkaline media on Pt	3.5×10^{-5}	$A\ cm^{-2}$	9,10
$i_{0,\mathrm{OER,acid}}$, exchange current density of OER in acidic media on Pt	1.3×10^{-11}	$A\ cm^{-2}$	11
$i_{0,\mathrm{OER,base}}$, exchange current density of OER in alkaline media on Pt	3.8×10^{-11}	$A\ cm^{-2}$	12
$\alpha_{\mathrm{HER,acid}}$, transfer coefficient of HER in acidic media on Pt	0.80	-	9,10
$\alpha_{\mathrm{HER,base}}$, transfer coefficient of HER in alkaline media on Pt	0.39	-	9,10
$\alpha_{\mathrm{HER,acid}}$, transfer coefficient of OER in acidic media on Pt	0.66	-	11
$\alpha_{\mathrm{HER,acid}}$, transfer coefficient of OER in alkaline media on Pt	0.88	-	12
$E^{\theta}_{\mathrm{HER,acid}}$, thermodynamic potential of HER in acidic media	0	V	13
$E^{\theta}_{\mathrm{HER,base}}$, thermodynamic potential of HER in alkaline media	-0.83	V	13
$E^{\theta}_{\mathrm{OER,acid}}$, thermodynamic potential of OER in acidic media	1.23	V	13
$E^{\theta}_{\mathrm{OER,base}}$, thermodynamic potential of OER in alkaline media	0.4	V	13

Supplementary Reference

1. Huang, J. Chen, Y. Eikerling, M. Correlated surface-charging behaviors of two electrodes in an electrochemical cell. *Proceedings of the National Academy of Sciences* **120**, e2307307120 (2023).
2. Onsager, L. Deviations from Ohm's law in weak electrolytes. *The Journal of chemical physics* **2**, 599-615 (1934).
3. Strathmann, H. Krol, J. J. Rapp, H. J. Eigenberger, G. Limiting current density and water dissociation in bipolar membranes. *Journal of Membrane Science* **125**, 123-142 (1997).
4. Esposito, D. V. Membraneless electrolyzers for low-cost hydrogen production in a renewable energy future. *Joule* **1**, 651-658 (2017).
5. Lin, K. Li, Z. Tao, Y. Li, K. Yang, H. Ma, J. Sha, J. Chen, Y. Surface charge density inside a silicon nitride nanopore. *Langmuir*, **37**, 10521-10528 (2021).
6. Werkhoven, B. L. Everts, J. C. Samin, S. Van Roij, R. Flow-induced surface charge heterogeneity in electrokinetics due to stern-layer conductance coupled to reaction kinetics. *Physical review letters*, **120**, 264502 (2018).
7. Tanaka, Y. Water dissociation reaction generated in an ion exchange membrane. *Journal of Membrane Science* **350**, 347-360 (2010).
8. Agmon, N. Bakker, H. J. Campen, R. K. Henchman, R. H. Pohl, P. Roke, S. Thämer, M. Hassanali, A. Protons and hydroxide ions in aqueous systems. *Chemical reviews* **116**, 7642-7672 (2016).
9. Zheng, Y. Jiao, Y. Vasileff, A. Qiao, S. Z. The hydrogen evolution reaction in alkaline solution: from theory, single crystal models, to practical electrocatalysts. *Angewandte Chemie International Edition* **57**, 7568-7579 (2018).
10. Schmidt, T. J. Ross Jr, P. N. Markovic, N. M. Temperature dependent surface electrochemistry on Pt single crystals in alkaline electrolytes: Part 2. The hydrogen evolution/oxidation reaction. *Journal of Electroanalytical Chemistry* **524**, 252-260 (2002).
11. Carmo, M. Fritz, D. L. Mergel, J. Stolten, D. A comprehensive review on PEM water electrolysis. *International journal of hydrogen energy* **38**, 4901-4934 (2013).
12. Zeng, K. Zhang, D. Recent progress in alkaline water electrolysis for hydrogen production and applications. *Progress in energy and combustion science* **36**, 307-326 (2010).
13. Ding, Y. Cai, P. Wen, Z. Electrochemical neutralization energy: from concept to devices. *Chemical Society Reviews* **50**, 1495-1511 (2021).
